# Rheological Characterization of Next-Generation Ballistic Witness Materials for Body Armor Testing

**DOI:** 10.3390/polym11030447

**Published:** 2019-03-08

**Authors:** Ran Tao, Kirk D. Rice, Anicet S. Djakeu, Randy A. Mrozek, Shawn T. Cole, Reygan M. Freeney, Aaron M. Forster

**Affiliations:** 1 Material Measurement Laboratory, National Institute of Standards and Technology, Gaithersburg, MD 20899, USA; kirk.rice@nist.gov; 2 Department of Chemical Engineering, Texas Tech University, Lubbock, TX 79409, USA; 3 Department of Chemical and Biomolecular Engineering, University of Maryland, College Park, MD 20740, USA; adjakeus@umd.edu; 4 U.S. Army Research Laboratory, Aberdeen Proving Ground, MD 21005, USA; randy.a.mrozek.civ@mail.mil (R.A.M.); shawn.t.cole7.civ@mail.mil (S.T.C.); 5 U.S. Army Aberdeen Test Center, Aberdeen Proving Ground, MD 21005, USA; reygan.m.freeney.civ@mail.mil

**Keywords:** rheology, polymer composites, rubber process analyzer, ballistic clay, LAOS, viscoplasticity, viscoelasticity

## Abstract

Roma Plastilina No. 1 (RP1), an artist modeling clay that has been used as a ballistic clay, is essential for evaluation and certification in standards-based ballistic resistance testing of body armor. It serves as a ballistic witness material (BWM) behind the armor, where the magnitude of the plastic deformation in the clay after a ballistic impact is the figure of merit (known as “backface signature”). RP1 is known to exhibit complex thermomechanical behavior that requires temperature conditioning and frequent performance-based evaluations to verify that its deformation response satisfies requirements. A less complex BWM formulation that allows for room-temperature storage and use as well as a more consistent thermomechanical behavior than RP1 is desired, but a validation based only on ballistic performance would be extensive and expensive to accommodate the different ballistic threats. A framework of lab-scale metrologies for measuring the effects of strain, strain rate, and temperature dependence on mechanical properties are needed to guide BWM development. The current work deals with rheological characterization of a candidate BWM, i.e., silicone composite backing material (SCBM), to understand the fundamental structure–property relationships in comparison to those of RP1. Small-amplitude oscillatory shear frequency sweep experiments were performed at temperatures that ranged from 20 °C to 50 °C to map elastic and damping contributions in the linear elastic regime. Large amplitude oscillatory shear (LAOS) experiments were conducted in the non-linear region and the material response was analyzed in the form of Lissajous curve representations with the values of perfect plastic dissipation ratio reported to identify the degree of plasticity. The results show that the SCBM exhibits dynamic properties that are similar in magnitude to those of temperature-conditioned RP1, but with minimal temperature sensitivity and weaker frequency dependence than RP1. Both SCBM and RP1 are identified as elastoviscoplastic materials, which is particularly important for accurate determination of backface signature in body armor evaluation. The mechanical properties of SCBM show some degree of aging and work history effects. The results from this work demonstrate that the rheological properties of SCBM, at small and large strains, are similar to RP1 with substantial improvements in BWM performance requirements in terms of temperature sensitivity and thixotropy.

## 1. Introduction

The material properties of ballistic witness materials (BWMs), particularly in response to high rate impact, play an important role for the assessment of personal protective systems such as body armor and helmets that are used in day-to-day activities for law enforcement officers and military personnel. During a ballistic resistance test, BWMs are used as a backing support for the protective equipment and, by virtue of their plastic response, indicate the behind-armor backface signature (BFS) after the ballistic event. The BFS evaluation is an important measurement as it provides information on the behind-armor effects that are indicative of the energy transferred to the human torso through the armor upon impact from a non-perforating projectile [1,2,3]. The initial research by Prather et al. [4] related the BFS, in ballistic gelatin and two types of clay, with behind-armor effects. Roma Plastilina No. 1 (RP1) was reported to have comparable ballistic performance as the gelatin with the advantage that the residual cavity formed in RP1 was easily measured and could provide a value for the BFS. Consequently, the National Institute of Justice (NIJ) and the U.S. Army adopted RP1 as the standard BWM for body armor [1,5,6]. While the performance standards for body armor have undergone periodic revisions, RP1 is still the required BWM [1,6,7].

RP1 is an oil-based artist clay that is a multicomponent, multiphase formulation composed of clay minerals, oils, waxes, fillers, and additives, with the specific composition of each constituent unspecified [2,3,8]. RP1 is known to show thixotropic characteristics with mechanical properties depending on time and work history. For example, RP1 becomes softer after physical manipulation (“working”) and hardens after the cessation of working over time. RP1 also exhibits temperature-dependent properties [8,9,10,11]. The thixotropy and temperature sensitivity of RP1 potentially introduce undesired variation in BFS measurements. Over the decades, since RP1 was adopted as the BWM standard, the RP1 formulation has changed to accommodate the demands of the artist community rather than BWM performance requirements [2]. Newer versions of RP1 are stiffer at room temperature than the original RP1, which necessitates modifications by the standards testing community to modify the clay handling procedure to meet calibration and verification specifications for clay performance. The standards community addressed these changes by incorporating a verification procedure for the RP1 clay prior to ballistic testing called a “drop calibration test”, which involves dropping an impactor of given mass and geometry from a specified height onto the surface of the clay block and then verifying that the indentation depth is within specification limits [12,13]. As the RP1 formulation has changed, thermal conditioning has been added to meet this calibration requirement. Currently, the clay block is heated to 100 °F (~37.8 °C) or higher [12] to pass calibration. The unspecified formulation, complex thermomechanical behavior, temperature sensitivity of RP1, together with the extra thermal conditioning procedure, have led to an initiative to develop an alternative room-temperature backing material to replace RP1, as recommended in the National Research Council (NRC) reports [2,3]. The major requirements specified in these reports for the alternative BWM are that it possesses specified, predictable, and controllable properties, with minimal changes to the current calibration protocols for RP1 and BFS test standards for protective equipment.

The Army Research Laboratory (ARL) has developed a room-temperature silicone composite backing material (SCBM) as a candidate material to replace RP1 [14,15]. The family of SCBMs is a particle-loaded polymer-based composite that consists of three components, i.e., non-crosslinked linear polydimethylsiloxane (PDMS), fumed silica, and corn starch [15]. The PDMS has a molecular weight that is slightly more than the entanglement molecular weight, such that elastic recovery is minimized. The fumed silica is added to the non-crosslinked PDMS liquid as a thickener to provide dimensional stability [16,17], and the corn starch serves as a detackifier. This three-component system is anticipated to meet most criteria for BWM, including the minimal number of components and readily tunable formulation, controllable mechanical properties, nontoxicity, low adhesion to the armor, minimal odor, low cost, minimal temperature sensitivity, and reusability at room temperature [14]. Edwards et al. have shown that the SCBM exhibits similar room temperature properties compared to heated RP1 by conducting side-by-side tests including quasi-static indentation and compression tests, drop tests, and body armor ballistic resistance tests [15]. While these previous studies provide correlation between the quasi-static and dynamic performance of SCBM and RP1, it is difficult to classify the deformation behavior of SCBM and RP1 as a function of strain, strain rate, and temperature.

Rheology is an important type of material characterization that reveals a wealth of valuable information on the response of a material to strain, temperature, and time [18,19]. This research employs rheology as an effective tool to characterize viscoelastic and viscoplastic properties of SCBM and RP1 over a wide temperature range, as well as to quantify the effects of aging and work history on rheological properties. To date, no studies have been reported on the comparison between the mechanical properties of these materials to understand whether the temperatures, strains, and strain rates of the two material classes are similar or different. This paper reports valuable material data on the new backing materials developed by the U.S. Army, which is important to ballistic researchers and the standards-based body armor testing community. This work supplements prior quasi-static indentation and ballistic resistance studies [15] for a complete characterization on SCBM properties and RP1 under shear deformation at low strain rates. Additionally, this work highlights the effectiveness of rheological techniques to help optimize formulations, to set quality control guidelines for RP1 and any candidate replacement material, as well as to establish documentary performance standards and specifications related to next-generation BWMs for body armor testing. In this work, dynamic oscillatory shear tests are performed at small strains, in which the applied strain varies sinusoidally with time, and the resulting shear stress is measured to quantify the elastic (*G*’) and viscous (*G*’’) components of the complex shear modulus (G*). Large amplitude oscillatory shear (LAOS) experiments [20,21] are performed in the nonlinear viscoelastic regime by variation of strain amplitude (strain) and frequency (strain rate) of the imposed sinusoidal oscillation. We compare the responses of SCBM at room temperature with the heated RP1, as well as the results for each material before and after aging and hand working. Finally, we report a scaling relationship of the storage modulus to high-rate deformations in these BWMs and discuss structure–property relationships in SCBM.

## 2. Materials and Methods 

### 2.1. Materials

RP1 clay was used as received from the manufacturer (Sculpture House, Springhill, NJ, USA). The SCBMs were produced by the ARL (Aberdeen Proving Ground, MD, USA) [14,15]. Three formulations of SCBM containing mass fractions of fumed silica that ranged from 13% to 18% by mass and were designated as SCBM #1, SCBM #2, and SCBM #3 were investigated. The material preparation procedure and processing parameters have been described in detail in Edwards et al. [15]; here, we briefly describe the SCBM preparation method for the sake of completeness. Untreated fumed silica, PDMS (with a low glass transition temperature near −120 °C), and corn starch were weighed out and pre-mixed using a commercial countertop stand mixer until the material became homogeneous by visual inspection. Then, the mixture was further compounded using a laboratory twin-screw extruder to produce the final product.

### 2.2. Methods

Rheological experiments were performed using a rubber process analyzer (RPA) (RPA *elite* from TA Instruments, New Castle, DE, USA) with an enhanced cooling system that employs pressurized air to cool the test chamber. The RPA is a strain-controlled rotational shear rheometer for rubber testing [22]. The RPA is advantageous for measuring BWMs due to a higher torque range (up to 25 N·m) for solid materials that enables access to large shear strain regimes. Second, it provides a consistent sample loading procedure, as ensured by the pressure pneumatic system together with the automated gap closure for sample sealing, such that variations in the measurement due to loading effects from specimen to specimen are minimized. The RPA is equipped with radial serrated bi-cone-shape platens with a fixed gap of 0.48 mm. The die surface serrations are designed for a constant sample contact at all strain amplitudes. The conversion from strain angle in degree to strain is: strain = *K*_γ_·strain angle, where the strain constant *K*_γ_ is 0.13655 (1/degree).

#### 2.2.1. Specimen Preparation

Each specimen was cut from the as-received extrudate into a ~70-mm-thick rectangular parallelepiped using a stiff scalpel blade and trimmed to achieve a mass of ~5 g to ensure consistency between specimens placed into the RPA. The test specimen was then placed between two sheets of polyester film and loaded onto the lower die of the instrument for measurements. The polyester film was used for ease of test specimen release and die cleaning between experiments. To ensure that no additional loading effects or thermal history influenced the measurement, the same loading procedure was applied, which was using a fresh specimen for each test, loading the specimen at the test temperature, and allowing the specimen to reach thermal equilibrium for 30 min prior to measurements.

#### 2.2.2. Frequency Sweep Experiments

Frequency sweep experiments were performed at a 0.14% strain (equivalent to a 0.01° strain angle), within the linear viscoelastic regime, from 0.05 Hz to 50 Hz at 5 points per decade, at five temperatures, i.e., 20 °C, 25 °C, 30 °C, 40 °C, and 50 °C. The test temperatures covered the operating temperature range expected for current RP1 calibration and testing. A new specimen was used for each test. The data for storage modulus and loss modulus were obtained and analyzed. For these experiments, the uncertainties in storage and loss moduli were approximately 3% and 10%, respectively.

#### 2.2.3. Large Amplitude Oscillatory Shear Experiments

Large amplitude oscillatory shear (LAOS) experiments were carried out at six frequencies of nominally 0.016 Hz, 0.063 Hz, 0.25 Hz, 1 Hz, 4 Hz, and 16 Hz each at 25 °C and 40 °C. Due to the limitation of the maximum shear rate (50 s^−1^), together with the amplitude limits (0.005° to 360°), which corresponds to strain limits (0.07% to 5000%) in the RPA bi-cone geometry, the frequency and strain ranges were judiciously chosen to cover a wide experimental test window. For example, at 0.016 Hz, the strain amplitude ranged from 0.07% (equivalent to a 0.005° strain angle) to 4394% (equivalent to a 315° strain angle); while at 1 Hz, the strain ranged from 0.07% (equivalent to 0.005° strain angle) to 697% (equivalent to 50° strain angle). The strain amplitudes were selected to cover 10 points per decade of the predetermined range. At each frequency, 1 pre-cycle was applied and a total of 10 cycles were measured for LAOS experiments. The data for stress and strain were recorded as a function of time. Since the presence of higher-order harmonics is a more sensitive indicator of the onset of nonlinearity compared to the decrease in *G*’ [23], we defined the linear viscoelastic limit as the strain amplitude, within which the relative intensity of the third harmonics (*I*_3_/*I*_1_) from the Fourier analysis of the oscillatory stress waveform was less than 0.03.

#### 2.2.4. Aging and Work History Effects

The effects of aging and work history are important to the properties of BWMs. We used two methods to isolate these effects for RP1 and SCBM. In order to investigate aging, samples from the small-strain frequency sweep test (strain = 0.14% and T = 25 °C) were left in their post-measured (pressed) form between the polyester films for five months in ambient conditions to simulate an exaggerated aging protocol where the BWM was prepared and then left for later use or study. After the aging time, samples were loaded into the RPA using the serrated plate indentations to align the film with the original orientation. The small-strain oscillatory behavior was re-measured using a frequency sweep test at the same conditions (strain = 0.14% and T = 25 °C).

The following procedure was followed to study the effect of work history. The materials were worked by hand for five minutes, in a way similar to kneading bread dough such that any transient structures should be disrupted [8]. The worked materials were then cut into blocks and loaded into the RPA applying the same frequency sweep test procedure, which used a strain of 0.01° and a frequency range from 0.05 Hz to 50 Hz. Care was taken to minimize the introduction of air into the materials. Three measurement repetitions were conducted on each sample.

## 3. Results and Discussion

### 3.1. Frequency Sweep Experiments

**Figure 1:**
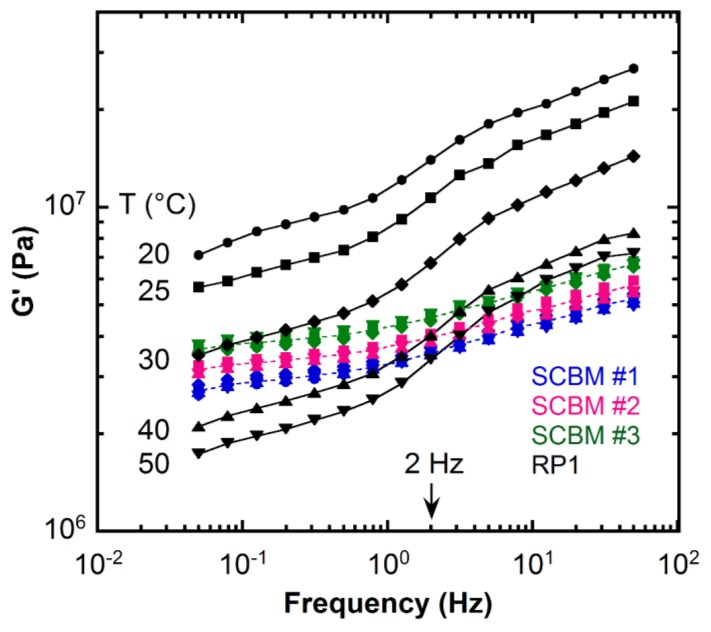
Dynamic shear storage modulus (*G*’) as a function of frequency measured at a strain of 0.14% (within the linear range) at different temperatures ranging from 20 °C to 50 °C for the SCBM materials and the RP1 clay [11]. The data points are color-coded based on different materials: blue—SCBM #1; pink—SCBM #2; green—SCBM #3; black—RP1 clay. Different symbols represent data points at different temperatures for each material: circle ●—20 °C; square ■—25 °C; diamond ♦—30 °C; up-pointing triangle ▲—40 °C; down-pointing triangle ▼—50 °C. The solid lines for the RP1 clay are guides for the eye. The dashed lines are average *G*’ values from different temperatures for the SCBM materials. The measurement uncertainty is 3%. View in color for best clarity.

The dynamic shear storage modulus (*G*’) as a function of frequency for the SCBMs and the RP1 clay are shown on a double logarithmic plot in Figure 1. The corresponding loss modulus (*G*’’) is reported in Supplementary Materials (Figure S1). All of the materials exhibit *G*’ > *G*’’ over this frequency range, indicating viscoelastic solid-like behavior. As expected, the storage modulus of the RP1 clay shows a strong temperature dependence, i.e., *G*’ decreases as temperature increases [11]. For example, at 2 Hz, *G*’ decreases from approximately 14 MPa at 20 °C to about 3 MPa at 50 °C for RP1. The storage modulus of RP1 clay shares a similar shape at different temperatures. As frequency decreases, *G*’ decreases with a rapid drop in the range of 10 Hz to 1 Hz, followed by a more moderate decrease in the range of 1 Hz to 0.05 Hz. Comparatively, the storage moduli of SCBM at different temperatures almost collapse into a single curve for each mass fraction, with the trend of *G*’ being SCBM #3 > SCBM #2 > SCBM #1, indicating that SCBMs are temperature-insensitive within the measured temperature range. The shear modulus trend of the SCBMs agrees with previous quasi-static hemispherical penetration force results on these SCBMs [14,15], which showed fumed silica loading as the dominant contributor to SCBM stiffness. We use the *G*’ value at 2 Hz to compare the different materials rather than the average *G*’ over the whole frequency range throughout the paper. The storage moduli of SCBM and RP1 at 2 Hz are reported in Table 1.

The current RP1 formulation is conditioned at or above 100 °F (approximately 38 °C) to satisfy the clay drop calibration test requirement [1]. For the RP1 clay at 40 °C, the storage modulus ranges from 2.1 MPa to 8.3 MPa over the frequency range from 0.05 Hz to 50 Hz, respectively, which establishes lower and upper bounds for the modulus that may be expected when RP1 is packed into the preform mold for calibration and testing. A remarkable finding is that the *G*’ data for all three SCBMs fall perfectly into this RP1 limit with a weaker frequency dependence across a 30 °C temperature range. A similar trend was observed for quasi-static penetration, compression, and impact tests at low strains and strain rates by Edwards et al. [15]. The ability of rheology to discern the differences in *G*’ for the three SCBM’s suggests a quantitative method that may be combined with ARL’s quasi-static force penetration experiments to standardize the formulation of candidate BWMs [14,15].

### 3.2. Large Amplitude Oscillatory Shear 

**Figure 2:**
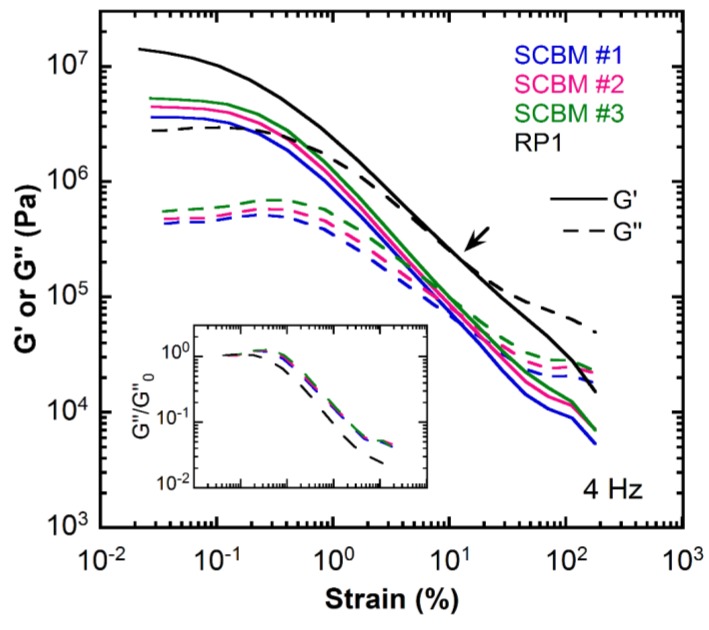
Dynamic shear storage modulus (*G*’, solid lines) and loss modulus (*G*’’, dashed lines) as a function of strain amplitude measured at 4 Hz and 25 °C for the SCBM materials and the RP1 clay. The arrow indicates the crossover strain (strain at *G*’ = *G*’’) for the RP1 clay. The inset figure shows reduced loss modulus (*G*’’/*G*_0_’’ as dashed lines, where *G*_0_’’ is taken as the loss modulus at the smallest strain) as a function of strain amplitude. Colors are the same as in Figure 1. The measurement uncertainty in *G*’ and *G*’’ data are ±3% and ±10%, respectively. View in color for best clarity.

Representative results from large-strain sweep experiments measured at 4 Hz and 25 °C are shown in Figure 2. The examination of *G*’ and *G*’’ data at a fixed frequency is the easiest and most direct method to investigate non-linear viscoelasticity without obtaining raw oscillatory stress data. The same trend in the *G*’ magnitude observed in the frequency sweep data (Figure 1) is found in the strain sweep data (Figure 2) with the RP1 clay being the stiffest at room temperature, followed by SCBM #3, SCBM #2, and SCBM #1. For the RP1 clay, at small strains, the storage modulus exhibits monotonically decreasing behavior [11], whereas the SCBM materials show a broader linear viscoelastic regime in which the modulus is independent of strain. For all of the materials, the storage modulus exceeds the loss modulus at small strains, indicating dominant elastic behavior. As the strain increases, *G*’ and *G*’’ decrease, eventually intersecting at a crossover strain, as shown by the arrow in Figure 2 for the RP1 clay, and beyond this, strain *G*’’ exceeds *G*’, indicating a dominant viscous response.

**Figure 3:**
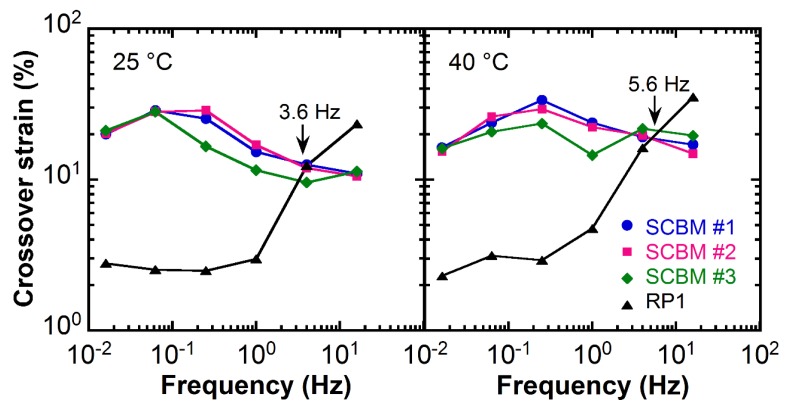
The crossover strains of *G*’ and *G*’’ from amplitude sweep measurements at 25 °C (**left panel**) and 40 °C (**right panel**) as a function of frequency. View in color for best clarity.

The crossover strain obtained from the strain sweeps is plotted against frequency in Figure 3. The data from measurements at 25 °C are shown on the left panel, and data for 40 °C are shown on the right panel. The intersection frequency, *f*_co_, of *G*’ and *G*’’ is often of practical importance since for *f* > *f*_co_, the material displays viscoelastic liquid-like properties; whereas for *f* < *f*_co_, the material behaves as an elastic solid. At low frequencies, i.e., frequencies of <3.6 Hz for the 25 °C data and frequencies of <5.6 Hz for the 40 °C data, the crossover strain for the SCBM materials are almost one order of magnitude higher than that of the RP1 clay. This indicates that the RP1 clay is a better dissipative system at low frequencies, i.e., the clay requires a lower strain energy or work to transition into a viscous liquid-like material that dissipates energy. On the contrary, at higher frequencies, the SCBM samples become a better damping material.

For both of the clay and SCBM materials, the shear modulus decreases as strain increases (Figure 2). However, an interesting observation with increasing strain is that *G*’’ shows a plateau followed by a weak strain overshoot and then a decrease (inset in Figure 2) while *G*’ is decreasing. Hyun et al. characterized this as a weak strain overshoot non-linear behavior [24], which is one type of the four basic LAOS behaviors that can be used to classify complex fluids based on the interaction between microstructures subjected to large deformations. The inset of Figure 2 shows the reduced loss modulus (*G*’’/*G*_0_’’) as a function of strain amplitude normalized with respect to the linear viscoelastic value *G*_0_’’ (taken as the loss modulus at the smallest measured strain) of each material. The reduced loss moduli at other frequencies, together with the data for the RP1 clay measured at 40 °C, are shown in Supplementary Materials (Figure S2). The reduced storage and loss moduli data of RP1 at 40 °C share a similar overall shape with those measured at 25 °C; this is in agreement with findings from our previous report [11], in which the normalized complex modulus curves at different temperatures were found to collapse into a single curve, suggesting that there is no significant thermal transition or structural change that affects the strain dependence of the shear modulus in the measured temperature range. The same findings are also observed for the SCBM materials. The overshoot in *G*’’ of the SCBM materials is found to be more pronounced than that of the RP1 clay at 4 Hz. The weak strain overshoot responses are often associated with systems having long side chains [24,25,26], sensitive filler networks [27,28] or suspended particles [29], large-scale structural rearrangement [30], or slight reorganization that offers additional dissipation [31,32]. Those complex microstructures resist flow alignment as the strain increases to a certain degree (*G*’’ increases), and then break down or align with the flow under large deformation, exhibiting strain thinning behavior (*G*’ and *G*’’ decrease).

This weak strain overshoot behavior in the non-linear region shows a frequency dependence (Figure S2, Supplementary Materials) for the materials investigated, i.e., the overshoot in *G*’’ is absent at the lowest frequency (0.016 Hz) for the SCBM materials and observed only at 4 Hz and 16 Hz for the RP1 clay, and it is more pronounced at high frequencies (i.e., at 4 Hz and 16 Hz) for both materials. The same frequency-dependent nonlinear behavior, i.e., strain thinning at low frequencies and weak strain overshoot at high frequencies, is also observed in associative polymers [25,26]. Sim et al. [33] predicted this behavior using a network model where the loss rate of the network junction is faster than the creation rate with both rates increasing with strain amplitude. In other cases [23,31], the maximum of *G*’’ overshoot decreases with increasing frequency. For this study, the weak strain overshoot may be attributed to structural changes that are caused by destruction and reformation of hydrogen bonding interactions between the PDMS chains and the starch granules [34] or unstable silica clusters [27] within the SCBM materials, and by the transient network structures in the RP1 clay [8]. The differences in the nonlinear frequency and strain behavior observed in different complex materials bring the need for quantitative investigation of the raw LAOS stress data, which will be discussed in the following section.

### 3.3. LAOS Analysis

**Figure 4:**
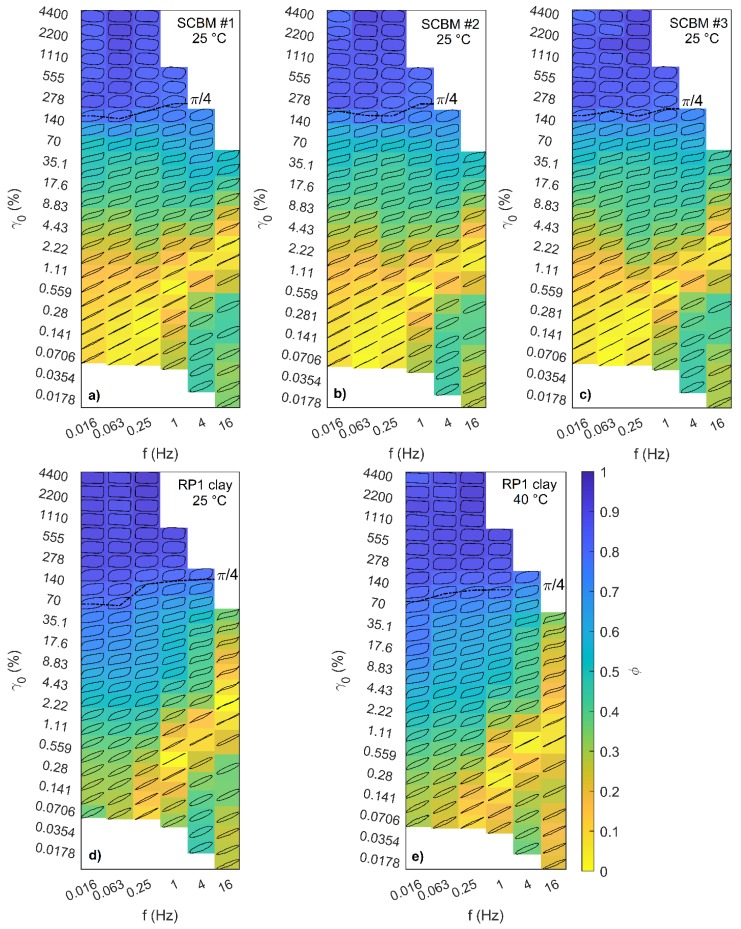
Tenth cycle of raw LAOS data for (**a**) SCBM #1, (**b**) SCBM #2, (**c**) SCBM #3, (**d**) RP1 clay measured at 25 °C, and (**e**) RP1 clay measured at 40 °C, shown as elastic Lissajous−Bowditch loops of normalized stress versus normalized strain (*σ*(*t*)⁄*σ*_max_ vs. *γ*(*t*)⁄*γ*_0_) within the Pipkin space of frequency and strain amplitude. The color filled within each Lissajous loop corresponds to the value of the dissipation ratio *ϕ* in the color bar of (e). The dashed line represents *ϕ* = *π*/4. View in color for best clarity.

Figure 4 shows the steady-state LAOS data as a set of the elastic Lissajous–Bowditch curves within the Pipkin space of {*f*, *γ*_0_}, where each curve is plotted as normalized stress versus normalized strain amplitude, {*σ*(*t*)/*σ*_max_} vs. *γ*(*t*)/ *γ*_0_, for all of the four materials investigated. Visual inspection of these patterns over the domain of the imposed strain amplitude and frequency are readily informative for the investigation of the overall linear to non-linear viscoelastic response. In the linear regime (small *γ*_0_), the response for a purely elastic solid material appears as a straight line on the elastic Lissajous curve, and that for a viscoelastic material appears as an ellipse. As *γ*_0_ increases, the curves become progressively distorted as the material enters the non-linear regime, and rich nonlinear behaviors are captured in the different non-elliptical distortions. Figure 4a–c show very similar patterns for the three SCBMs investigated. Differences in Lissajous patterns between SCBM and RP1 (Figure 4d,e) are now described. For the SCBM, at low frequencies and small strains {*f* < 0.25 Hz, *γ*_0_ < 0.3%}, the deformation is almost purely elastic as shown by linear Lissajous curves. As strain amplitude increases, the responses become nonlinear as reflected by the distortion of the ellipses that develop into rectangular shapes with strongly rounded corners. For the RP1 clay, at low frequencies and small strains (lower-left portion of the Pipkin space), the Lissajous curves exhibit elliptical shapes. The responses are rectangular at low frequencies and large strains {*f* = 0.016 Hz, *γ*_0_ = 4400%}, in contrast to those rounded-corner rectangles observed for SCBM at the same imposed LAOS condition.

In order to better quantify these transitions in non-linear behavior, we calculate the perfect plastic dissipation ratio *ϕ* which is introduced by Ewoldt et al. [35], defined as the energy dissipated in a single LAOS cycle, *E*_d_, divided by the energy dissipated for a perfect plastic response, (*E*_d_)_pp_, to investigate yielding response of these materials. *E*_d_ equals the area enclosed by the elastic Lissajous curve of stress versus strain, (*E*_d_)_pp_ = ∮*σ*(d*γ*), and (*E*_d_)_pp_ is the area of the rectangle for the corresponding perfect plastic response with strain amplitude *γ*_0_ and maximum stress *σ*_max_, i.e., (*E*_d_)_pp_ =(2*γ*_0_)(2*σ*_max_). This scalar parameter *ϕ* is a better indicator to identify yield-like behavior, i.e., distinguishing pseudoplastic liquids [36] from elastoviscoplastic materials, than other intra-cycle nonlinear measures such as the strain–stiffening ratio and the shear–thickening ratio [37], especially when strong nonlinearities are present [35]. When ϕ approaches 0, the response is purely elastic as no energy is dissipated; when ϕ approaches *π*/4 (~0.785), the response corresponds to a material that dissipates as much as energy as a Newtonian fluid, and when ϕ approaches 1, it uniquely corresponds to perfect plastic behavior and, at this deformation condition, the dynamic yield stress (*σ*_Y_) can be determined as *σ*_Y_ = *σ*_max_. The dissipation ratio ϕ is calculated and represented as the color filled within each Lissajous loop in Figure 4; also shown in Figure 4 are ϕ = *π*/4 lines. Both the SCBMs and RP1 show yield-like behavior as ϕ → 1. For the SCBMs, the maximum dissipation ratio is ϕ_max_ ≈ 0.92, which is observed at moderate frequencies and large strains, i.e., at {*f* = 0.063 Hz, *γ*_0_ = 4400%} for SCBM #1 and SCBM #2, and at {*f* = 0.25 Hz, *γ*_0_ = 4400%} for SCBM #3. The corresponding maximum stresses *σ*_max_ are 20.8 kPa, 23.4 kPa, and 40.2 kPa for SCBM #1, SCBM #2, and SCBM #3, respectively. For the RP1 clay, the maximum dissipation ratio ϕ_max_ ~ 0.95 occurs at {*f* = 0.25 Hz, *γ*_0_ = 4400%} with *σ*_max_ = 48.4 kPa.

A general description of the behavior for all materials, shown in Figure 4, is at small strains below the *ϕ* = *π*/4 line, the responses at deformations in the yellow colored regions of the Pipkin space are predominantly elastic; for the regions that are green colored, the responses are elastoplastic and dissipate energy less than that of a Newtonian fluid. As the strain increases, the dissipation ratio passes through the line of *ϕ* = *π*/4 and is colored blue, representing a highly yielded region in which the material behaves as a viscoplastic fluid. The corresponding Lissajous curves become increasingly rectangular as *ϕ* continues to increase and finally approach a nearly perfect plastic response.

From the colored Pipkin diagram, the differences between SCBM and RP1 are readily discerned. First, the yellow region is located mainly in the bottom-left corner (low frequency and small strain) of the Pipkin space for the SCBM materials, while it appears in the bottom-right region (high frequency and small strain) for RP1. This indicates that the SCBM is a more elastic material than the RP1 at small strain rates, in agreement with the results from Figure 3 where the RP1 is found to dissipate more energy than the SCBM at low frequencies. Second, the elastic-to-viscoplastic transition for the SCBM is higher than that of the RP1. For example, at 1 Hz, *ϕ* = *π*/4 occurs at *γ*_0_ = 258% for SCBM #1 and at *γ*_0_ = 144% for the RP1. The elastoplastic-to-viscoplastic transition is not assessed for frequencies higher than 1 Hz due to the limitations of the instrument (see Experimental). Nevertheless, during ballistic impacts involving much higher shear rates (of the order of 10^4^ s^−1^ to 10^5^ s^−1^), which would be located in the far top-right corner of the Pipkin space, both materials are expected to be in a highly yielded viscoplastic regime. In this regard, the SCBM materials satisfy one of the utmost important BWM requirements—viscoplasticity—by providing dimensional stability, deforming easily upon impact, and exhibiting minimal elastic recovery after deformation [15]. This is critical to achieve performance requirements for BWMs because for applied stresses below the yield stress, the BWM is desired to behave as a viscoelastic solid which is dimensionally stable; upon elevated applied stresses exceeding the yield stress, the BWM is expected to irreversibly deform and flow as a fluid with permanent deformation. Analysis on the nonlinearity was also performed, and the results for the relative intensity of the 3^rd^ harmonic component from Fourier analysis of the LAOS data (*I_3_*/*I*_1_) are shown in the same Pipkin space in Supplementary Materials (Figure S3). Differences in the nonlinear behavior for the RP1 clay and SCBM materials are revealed. For the SCBMs, the highest *I_3_* occurs in the center of the Pipkin space (intermediate strain and frequency); while for the RP1, the highest *I_3_* appears at low frequencies and large strains. Furthermore, for a comparison within the SCBM systems, SCBM #3 presents the highest nonlinearities as shown by the dark blue regions, which is as expected because nonlinear response is introduced by the addition of fumed silica with increasing filler volume fraction [38].

### 3.4. Aging and Work History Effects

**Figure 5:**
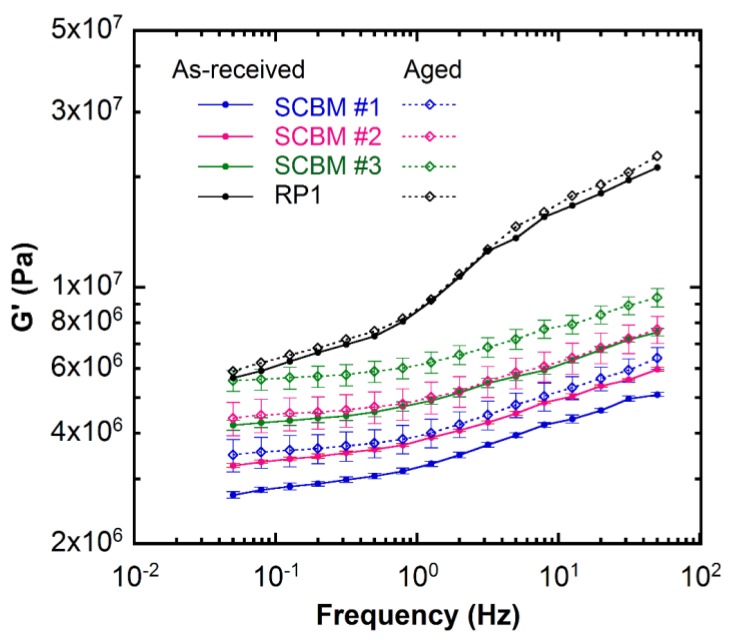
Dynamic shear storage modulus (*G*’) as a function of frequency measured at 25 °C for the as-received (solid circle ●) and aged (open diamond ◇) materials. The lines are guides for the eye. The error bars represent standard deviations of the mean from three separate measurements on different samples. View in color for best clarity.

The results of the aged (five months) materials from frequency sweep experiments at 25 °C are shown in Figure 5 as open symbols, along with the data for the as-received materials represented by solid symbols. The SCBMs become stiffer and the storage modulus *G*’ increases with aging time. The *G*’ values at 2 Hz for the aged and as-received materials are reported in Table 2. The increase in *G*’ is found to range from 21% to 28% for SCBMs. The exact origin of this stiffening behavior is not known, but may be explained by the changes in the filler network structure over time. As the material ages, more PDMS chains adsorb onto the surface of the fumed silica through hydrogen bonding, which leads to an increase in chain bridging between filler particles, and therefore resulting in network reorganization [16,39,40,41]. The stiffening may also be due to moisture-involved structures resulting from elevated moisture content [42], e.g., the increase in corn starch crystallinity [43]. Another finding from Figure 5 is that the variation in the *G*’ data for the aged materials increases compared to the as-received samples, e.g., the standard deviation from three separate measurements on different SCBM #2 samples increases from 1% to 10%. Based on the structural rearrangement hypothesis, the particle reagglomeration or filler network rearrangement during aging most likely introduces heterogeneity to the material structure, which produces larger standard deviation in the measured storage modulus for the aged material [38]. On the other hand, the storage modulus for the aged RP1 is similar to that of the as-received RP1 within the error of the measurements, suggesting that the re-organization of the structures in the RP1, if any, is a relatively slow process as compared to the SCBM at 25 °C.

**Figure 6:**
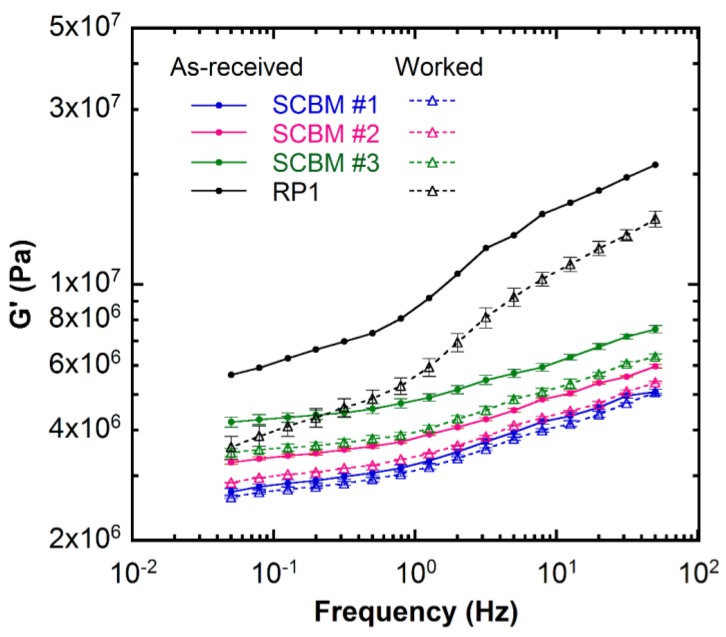
Dynamic shear storage modulus (*G*’) as a function of frequency measured at 25 °C for the as-received (solid circle ●) and worked (open triangles △) materials. The lines are guides for the eye. The error bars represent standard deviations of the mean from three separate measurements on different samples. View in color for best clarity.

Figure 6 shows a comparison between the worked and as-received materials measured from the same frequency sweep tests. The effect of work history is observed as a softening of the material and a reduction in the storage modulus for the worked materials as shown by open symbols. The *G*’ data for the worked materials are reported in Table 2. The decreases are 4%, 11%, and 17% for SCBM #1, SCBM #2, and SCBM #3, respectively. This decrease may be attributed to the breakdown of hydrogen bond interactions between the starch granules and the PDMS chains [34], as well as between the fumed silica surface and the PDMS chains [16,44,45], by hand working. Similarly, the worked RP1 sample also shows a modulus which is 35% lower than that of the as-received clay. RP1 is found to be more sensitive to work history than aging time, which may result from the multicomponent formulation that yields three-dimensional networks involving both microcrystalline and amorphous structures [8,46]. These transient networks can be easily disrupted by hand working. In practical use, the backing material is effectively worked on a regular basis during ballistic resistance testing of body armor, resulting in a combination of aging and work history. The shelf and performance lifetimes of the SCBM under expected usage conditions are currently being evaluated.

### 3.5. Scaling Relationship of Shear Modulus to Deformation at Higher Rates

**Figure 7:**
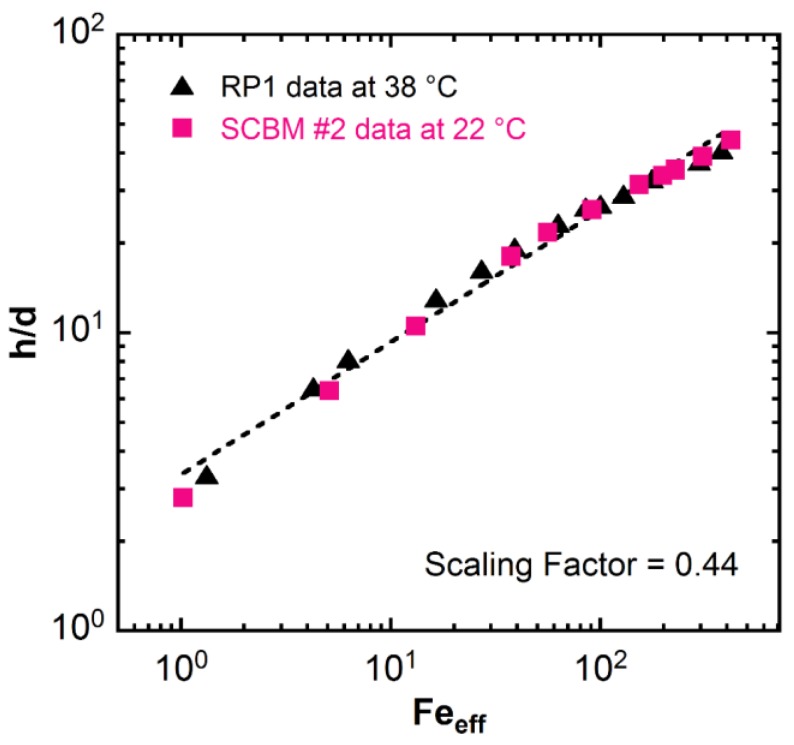
The unitless depth of penetration (*h*/*d*) as a function of the effective elastic Froude number *Fe*_eff_ for the RP1 at 38 °C and SCBM at 22 °C. Data are from Edwards et al. [15].

Measurements of mechanical properties at ballistic strain rates and strains are not a trivial task. Until standardized test methods are established that operate within this extreme deformation regime, there is a need to identify correlations of mechanical properties obtained at lower strain or strain rates with material responses under ballistic impact. Recent work from Mrozek et al. [47] identifies a correlation between mechanical properties and ballistic penetration. They reported a scaling relationship between the ballistic penetration depth and the effective elastic Froude number, *Fe*_eff_, in a viscoelastic triblock copolymer gel system, i.e., poly(styrene-b-ethylene-co-butylene -b-styrene) and mineral oil. The effective elastic Froude number correlates the density difference between the projectile and backing material, the effective projectile velocity, and the shear modulus of the backing material, i.e., *Fe*_eff_ = [(*ρ*_p_ − *ρ*_s_)*v*_eff_^2^/*G*] [47], where *ρ*_p_ is the density of the projectile, *ρ*_s_ is the density of the backing material, *v*_eff_ is the effective velocity which is the difference between the measured velocity *v* and the minimum velocity for penetration *v*_min_, *v*_eff_ = *v* − *v*_min_, and *G* is the shear modulus of the backing material. This type of analysis has not been conducted for the SCBM and RPI materials and we explore whether a scaling relationship exists between penetration depth and the mechanical properties reported here. Based on the ballistic penetration depth results on SCBM and RP1 from Edwards et al. [15], we apply the same plotting method, unitless depth of penetration as a function of effective Froude number in Figure 7. The values of *v*_min_ are determined by extrapolating the penetration depth vs. velocity curves to 0 mm depth, which are 18.1 m/s and 25.9 m/s for RP1 and SCBM #2, respectively. The shear modulus is taken as the storage modulus *G*’ at 2 Hz from Table 2. Interestingly, it seems to hold for the BWMs under investigation. The scaling factor obtained for all data is 0.44 ± 0.01, which is close to the observed 0.53 factor and the theoretical scaling of 0.5 reported by Mrozek et al. [47]. If this empirical relationship holds for RP1 and SCBM systems, then it is possible to estimate the effects of shear modulus on penetration depth. For example, a 25% increase in the shear modulus will lead to a 9% decrease in the penetration depth; a 15% decrease in the shear modulus results in a 7% increase in the penetration depth. The current acceptable experimental error in BFS is 10% of the maximum indentation depth. Therefore, the shear modulus may provide additional insight into the expected experimental error in BFS for a particular SCBM formulation. It should be noted that the relationship of shear modulus to ballistic performance is interesting but further study is required.

### 3.6. Strcture–Property Relationships in SCBM

The Army’s SCBM is a three-component system that is designed specifically for body armor testing applications. The formulation of non-crosslinked polydimethylsiloxane, fumed silica, and corn starch—all three of which are commercially available and cost-effective—is easily tunable through the component proportion to achieve controlled performance [15]. The characteristics of each constituent are important factors that influence the overall composite properties and material behavior. The structure–property relationship within fumed-silica-filled PDMS systems have been extensively explored by rheological studies [16,17,38,39,41,44,48,49]. Untreated fumed silica possesses a large volume of silanol groups on the silica surface as a result of the flame hydrolysis of silicon tetrachloride and the irreversible silica aggregates, with open fractal structures, that are generated from this fabrication process. Silica aggregates can be bridged to one another by PDMS chains through multiple hydrogen bonding between the hydroxyl groups on the silica surface and the oxygen atoms of the PDMS backbones [38,44,45], or they can form loose agglomerates connected by adhesion forces that can be easily broken down by aggressive shearing methods [50]. The interparticle interactions, via entanglements of the polymer adsorbed onto the silica surface, depend on the molecular weight of PDMS and silica loading [38,44], thus explaining complex rheological behavior of the fumed-silica-filled PDMS systems. For example, as the filler concentration increases, the probability for bridging through entanglements of polymer chains increases; therefore, the modulus increases and the linear viscoelastic region shortens. As the molecular weight of the polymer increases, the frequency dependence of the storage modulus increases because the strongly adsorbing layer of PDMS chains on the silica surface extends beyond the bound layer and interacts with other aggregates, and such bridging interactions are expected to be more frequency-dependent. Aging phenomenon in these systems have been investigated [39] and also found to depend on the PDMS molecular weight [16,40]. The structural evolution during aging depends on the interplay of the saturation of chain adsorption onto the filler surface and the formation of polymer bridging junctions, with the former shortening the effective network chains and resulting in filler agglomeration, while the latter weakens the interparticle attractions and increases the entanglement network density [16,40]. The incorporation of corn starch adds a further complication to the SCBM because its hygroscopic nature, i.e., corn starch and water mixture, are known to be shear-thickening, introducing nonlinearity and thixotropy for the mechanical responses. In brief, PDMS molecular weight and the loadings of fumed silica and corn starch can be considered as adjustable parameters for future optimization of SCBM formulation.

## 4. Conclusions

In this work, rheological studies were performed on a family of room-temperature SCBM, which were developed by the ARL as next-generation BWMs for body armor testing, as well as the current standard backing material, RP1, using a rubber process analyzer. Frequency sweep experiments were performed at different temperatures covering the operating temperature range for current RP1 validation. LAOS experiments were conducted and the data were analyzed in the form of Lissajous curve representations. Aging and work history effects on the rheological responses were also investigated.

Results obtained from the rheological measurements in the present work provide valuable information on the comparison between the RP1 and SCBM regarding the BWM requirements. The frequency sweep experiments at different temperatures demonstrate minimal temperature dependence for SCBM in a wide range of operational temperatures, nominally 20 °C to 50 °C. The values of the storage modulus for the SCBM at 25 °C are bounded by those of the RP1 at 40 °C, suggesting that the measurement of the shear modulus may be used as an alternative validation method for adjusting the formulation, in-situ process monitoring, and quality control of SCBMs. The weaker frequency dependence of the storage modulus observed for SCBMs implies that they exhibit less variation in material properties, which is beneficial for the candidate replacement material since this may improve repeatability in quasi-static, drop test calibrations, and ballistic calibration testing. From the strain amplitude sweep results, a weak strain overshoot in the loss modulus data is observed for the two materials, suggesting that structural changes occur upon deformation in these systems.

Viscoplasticity and yield stress behavior are particularly important for the backing material to obtain accurate BFS measurement. Such information is available from LAOS analysis by plotting Lissajous curves and examining the dissipation ratio. Lissajous curves provide a fingerprint of rheological nonlinearities for intuitive interpretation of transition from linear to non-linear responses in complex fluids [35]; in conjunction with the dissipation ratio calculation, LAOS signatures associated with yield stress fluids were identified and analyzed in the context of material deformation. The results show that the SCBM, similar to RP1, is an elastoviscoplastic material. The yield stress behaviors of these two materials differ in that the SCBMs display more elasticity at small deformation and have a lower yield stress, as compared to those of the heated RP1. Additionally, the study on aging and work history effects reveals information on the responses of RP1 and SCBM from a structure–property relationship perspective. The stiffening behavior upon aging may be attributed to the increase in the filler network density for the SCBM, or the moisture involved structures due to elevated moisture content. More work is required to determine whether the stiffening observed in the rheology has an effect on the ballistic response of the BWMs. Along with the study by Edwards et al. [15], the results obtained from this work support the SCBM as a promising room-temperature replacement material for the current standard backing material, RP1, for body armor validation.

## Figures and Tables

**Table 1:** The shear storage modulus (*G*’) at 2 Hz for the silicone composite backing materials (SCBMs) and Roma Plastilina No. 1 (RP1) from frequency sweep experiments at different temperatures.

	*G*’ at 2 Hz (MPa) ^a^					
Materials	T = 20 °C	T = 25 °C	T = 30 °C	T = 40 °C	T = 50 °C	Average ^b^
SCBM #1	3.55	3.53	3.68	3.52	3.45	3.55 ± 0.08
SCBM #2	3.98	4.57	4.07	3.85	3.86	4.07 ± 0.29
SCBM #3	4.45	4.62	4.46	4.59	4.67	4.56 ± 0.10
RP1	13.99	10.69	6.72	4.02	3.40	7.76 ± 4.51

^a^ Measurement uncertainties are 3%. ^b^ The average *G*’ value is taken as the average value of data at 2 Hz measured at five temperatures, i.e., 20 °C, 25 °C, 30 °C, 40 °C, and 50 °C. The standard deviations from these data are reported.

**Table 2:** The shear storage modulus (*G*’) at 2 Hz for as-received, aged, and worked SCBMs and RP1 from frequency sweep experiments at 25 °C.

	*G*’ at 2 Hz (MPa)				
	As-received	Aged		Worked	
Materials	Value	Value	Increased (%) ^a^	Value	Decrease (%) ^a^
SCBM #1	3.49 ± 0.06	4.23 ± 0.34	21	3.35 ± 0.04	4
SCBM #2	4.08 ± 0.03	5.21 ± 0.50	28	3.63 ± 0.01	11
SCBM #3	5.16 ± 0.14	6.55 ± 0.39	27	4.30 ± 0.08	17
RP1	10.69 ± 0.32	10.86 ± 0.40	2	6.95 ± 0.40	35

^a^ The changes are compared with the as-received samples measured at 25 °C.

## Supplementary Materials

The following are available online at https://www.mdpi.com/2073-4360/11/3/447/s1, Figure S1: Dynamic shear loss modulus (*G*’’) as a function of frequency, Figure S2: Reduced loss modulus (*G*’’⁄*G*_0_’’) as a function of strain amplitude at different frequencies, Figure S3: Last cycle of raw LAOS data for the SCBMs and the RP1 clay, shown as Lissajous−Bowditch loops of normalized stress versus normalized strain within the Pipkin space of frequency and strain amplitude.
