# Supplementary material for: Rheological Characterization of Next-Generation Ballistic Witness Materials for Body Armor Testing
Source: Polymers (Basel). 2019 Mar 8;11(3):447. doi: 10.3390/polym11030447 (PMC6473786; doi:10.3390/polym11030447)
Supplement: Supplementary file 1 [file polymers-11-00447-s001.pdf]

Article

Rheological Characterization of Next-Generation Ballistic Witness Materials for Body Armor Testing

Ran Tao [1,2,*], Kirk D. Rice [1], Anicet S. Djakeu [3], Randy A. Mrozek [4], Shawn T. Cole [4], Reygan M. Freeney [5] and Aaron M. Forster [1,*]

[1] Material Measurement Laboratory, National Institute of Standards and Technology, Gaithersburg, MD 20899, USA; kirk.rice@nist.gov
[2] Department of Chemical Engineering, Texas Tech University, Lubbock, TX 79409, USA
[3] Department of Chemical and Biomolecular Engineering, University of Maryland, College Park, MD 20740, USA; adjakeus@umd.edu
[4] U.S. Army Research Laboratory, Aberdeen Proving Ground, MD 21005, USA; randy.a.mrozek.civ@mail.mil (R.A.M.); shawn.t.cole7.civ@mail.mil (S.T.C.)
[5] U.S. Army Aberdeen Test Center, Aberdeen Proving Ground, MD 21005, USA; reygan.m.freeney.civ@mail.mil
* Correspondence: ran.tao@nist.gov (R.T.); aaron.forster@nist.gov (A.M.F.); Tel.: +1-301-975-2535 (R.T.); +1-301-975-8701 (A.M.F.)

Supplementary Materials

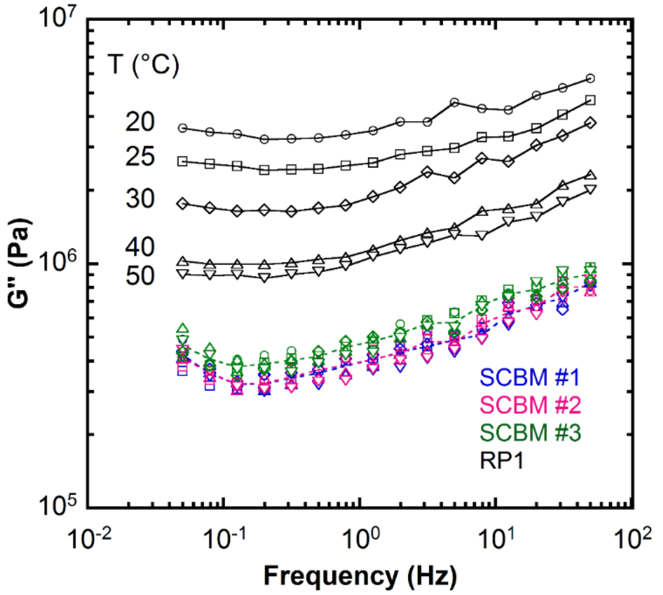

Figure S1. Dynamic shear loss modulus (*G''*) as a function of frequency measured at a strain of 0.14 % (within linear range) at different temperatures ranging from 20 °C to 50 °C for the SCBM materials and the RP1 clay. Experimental uncertainties in the data are ± 10 %. The color and shape for symbols and lines are the same as in Figure 1. View in color for best clarity.

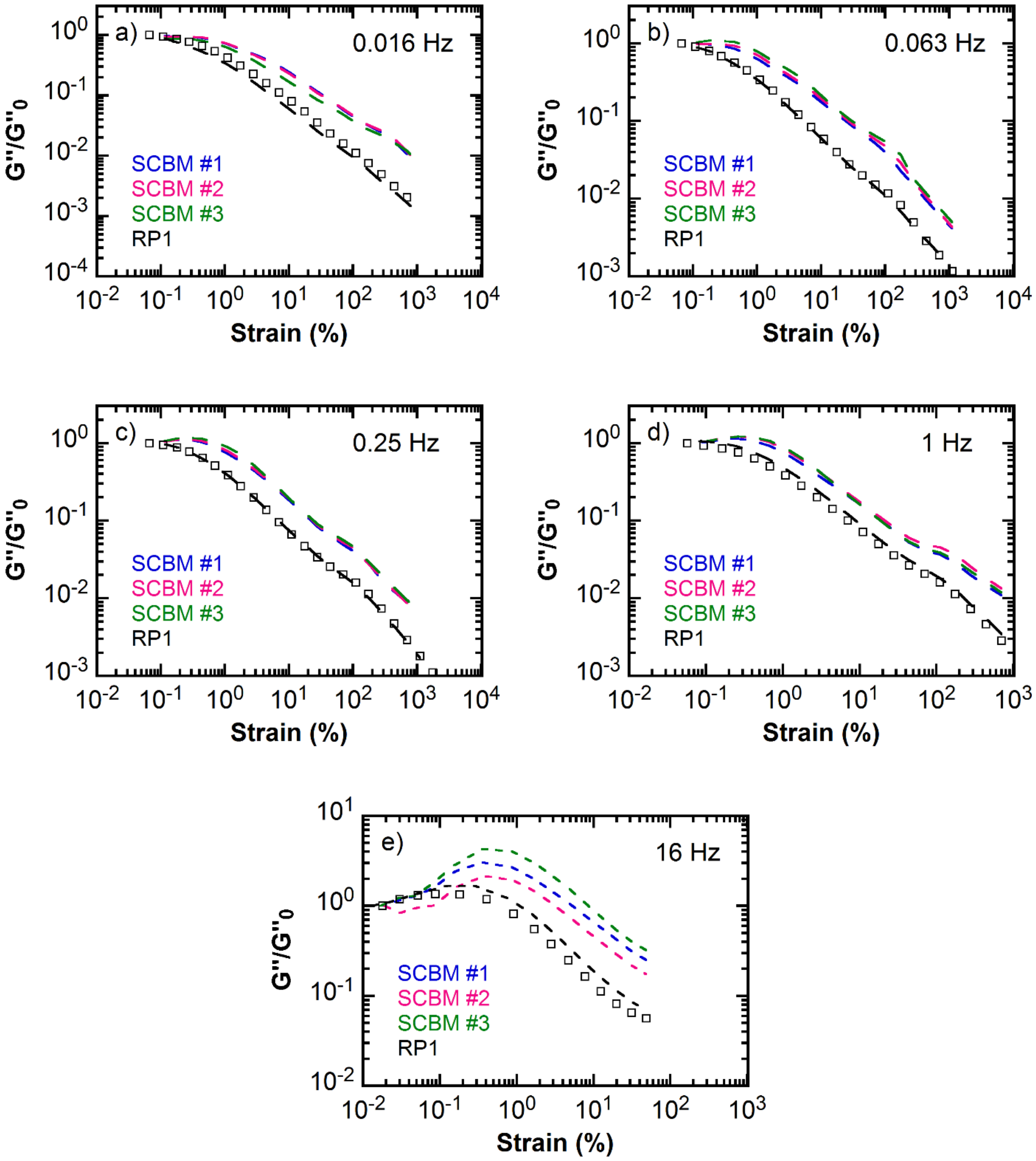

Figure S2. Reduced loss modulus (G''/G_0'') as dashed lines, both measured at 25 °C, where G_0'' is the linear viscoelastic modulus for each material as a function of strain amplitude at different frequencies of a) 0.016 Hz, b) 0.063 Hz, c) 0.25 Hz, d) 1 Hz, and d) 16 Hz. The data for the RP1 clay measured at 40 °C are also shown as open symbols. Experimental uncertainties in the data are ± 10 %. View in color for best clarity.

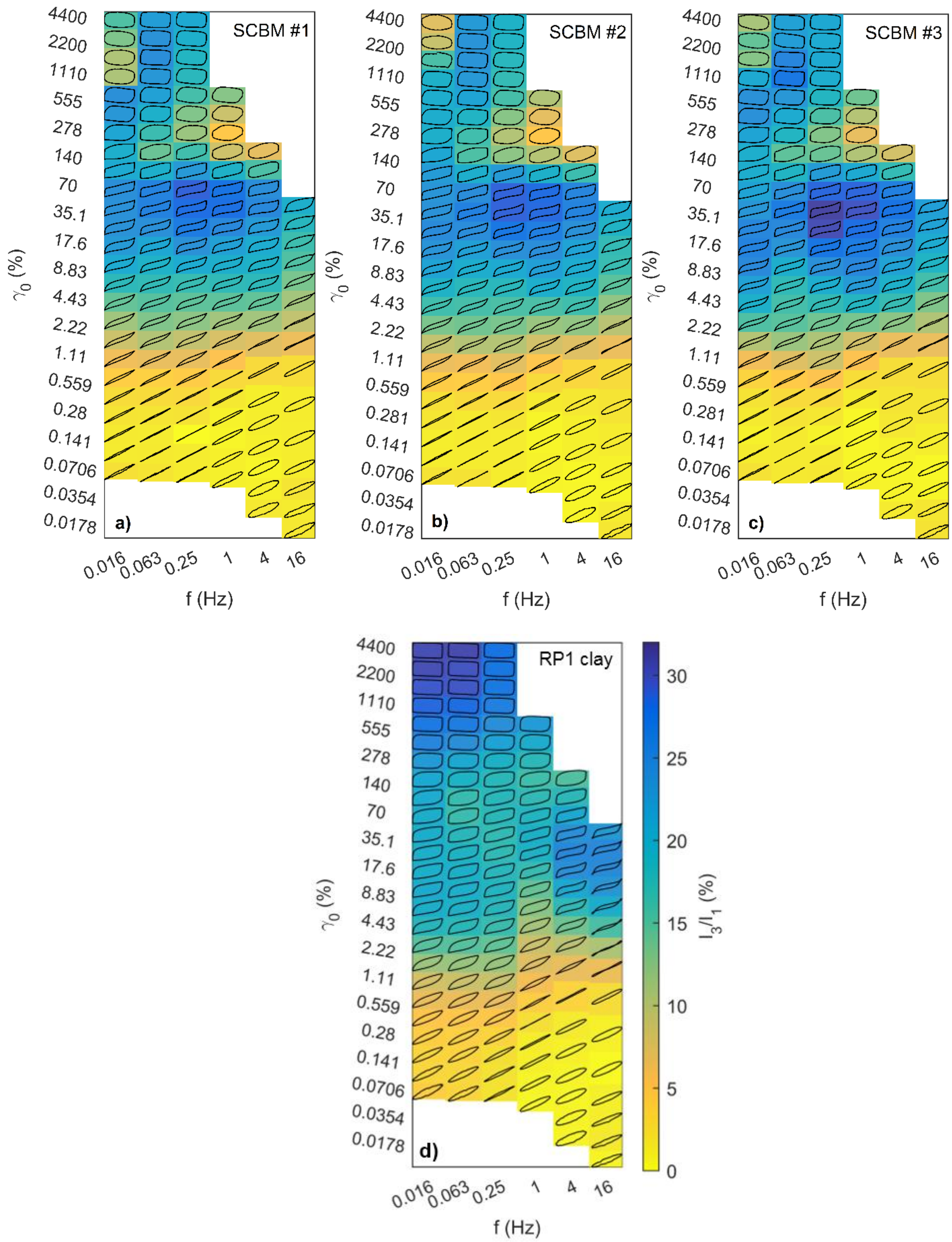

Figure S3. Last cycle of raw LAOS data for the SCBM materials and the RP1 clay, shown as Lissajous–Bowditch loops of normalized stress versus normalized strain within the Pipkin space of frequency and strain amplitude. The color filled within each Lissajous loop corresponds to the relative intensity of the third harmonics I_3/I_1 in the color bar of d). View in color for best clarity.
